# Cross‐species protein interactome network‐based analysis of GWAS and human brain quantitative trait loci (x‐QTL) data identifies risk genes and drug targets for Alzheimer’s disease

**DOI:** 10.1002/alz.092617

**Published:** 2025-01-03

**Authors:** Jielin Xu, Yadi Zhou, Yuan Hou, Andrew A. Pieper, Feixiong Cheng, James B Leverenz

**Affiliations:** ^1^ Cleveland Clinic, Cleveland, OH USA; ^2^ Louis Stokes VA Medical Center, Cleveland, OH USA; ^3^ Case Western Reserve University, Cleveland, OH USA; ^4^ Louis Stokes Cleveland VA Medical Center, Cleveland, OH USA; ^5^ University Hospitals Cleveland Medical Center, Cleveland, OH USA; ^6^ Cheveland Clinic Genome Center, Cleveland, OH USA; ^7^ Cleveland Clinic Lou Ruvo Center for Brain Health, Cleveland, OH USA

## Abstract

**Background:**

The emerging tools of protein‐protein interactome network offer a platform to explore not only the molecular complexity of human diseases, but also to identify risk genes and drug targets. Integration of the genome, transcriptome, proteome, and the interactome networks are essential for such identification, including Alzheimer’s disease (AD), Parkinson disease (PD), and Amyotrophic lateral sclerosis (ALS)

**Method:**

In this study, we performed multi‐modal analyses of cross‐species protein interactome networks and human brain functional genomics data to identify risk genes and drug targets for neurodegenerative diseases. We presented a multi‐view **t** opology‐based deep learning framework to identify disease‐**a** ssociated **g** enes for **cross**‐species interactome (TAG‐X). We re‐constructed comprehensive protein‐protein interactome networks for human, *Drosophila melanogaster* (fruit fly), *Caenorhabditis elegans* (worm), and *Saccharomyces cerevisiae*(yeast), by assembling high quality binary protein‐protein interactions (PPI). The fundamental premise of TAG‐X is that AD risk genes exhibit distinct functional characteristics compared to non‐risk genes and, therefore, can be distinguished by their aggregated human brain‐specific functional genomic features from various quantitative trait loci (x‐QTL), including expression QTL (eQTL), protein QTL (pQTL), splicing QTL (sQTL), methylation QTL (meQTL), and histone acetylation QTL (haQTL).

**Result:**

After integration genome‐wide association studies (GWAS) and x‐QTL data into the interactome networks via TAG‐X, we found that unique integration of fly, worm and yeast interactome networks boosted performance in risk gene prediction compared with the human protein‐protein interactome across AD (e.g., fly: *NDUFAF6, CHRNA2;* worm and yeast: *TOMM40*), PD (e.g., fly: *SCARB2*; worm: *AGAP1*; yeast: *SLC2A13*, *BCKDK*) and ALS (e.g., fly: *SOD1*; worm: *SARM1*; yeast: *VCP*, *PRDX6*). We found that human brain‐specific PPI network presented the strongest potential for AD risk gene discovery (e.g., AD: *ACE*, *BIN1*, *INPP5D*,*MS4A4A*, *SYK; PD: SNCA, LRRK2, DGKQ*; ALS: *SCFD1*, *G2E3*). Furthermore, interactome network‐predicted genes are significantly enriched in known drug targets and are significantly enriched in disease‐related pathobiological processes.

**Conclusion:**

In summary, we presented a cross‐species protein interactome network methodology that utilizes functional genomic and GWAS findings to identify disease risk genes and drug targets for AD and other neurodegenerative diseases if broadly applied. Functional observations of candidate targets and genes are warranted in the future.

